# Supplementary material for: Adherence to Anti-Osteoporotic Treatment and Clinical Implications after Hip Fracture: A Systematic Review
Source: J Pers Med. 2021 Apr 24;11(5):341. doi: 10.3390/jpm11050341 (PMC8146075; doi:10.3390/jpm11050341)
Supplement: Supplementary file 1 [file jpm-11-00341-s001.zip › jpm-1191565-supplementary.pdf]

Table S1. Study characteristics and limitations									
**Study, year**	**Limitations**								
	**Prospective study design**	**No design flaws**	**Large Number of patients***	**Data regarding treatment**	**Data regarding adherence/compliance**	**Patient confounders**	**Confounders related to treatment**	**Follow-up >2 years**	**Significant results (p<0.05)**
Behanova *et al.*, 2019 [13]	x	ν	ν	x	x	x	x	ν	ν
Bondo *et al.*, 2013 [14]	x	ν	ν	x	ν	ν	x	ν	ν
Bergman *et al.*, 2019 [15]	x	ν	ν	ν	x	ν	x	ν	ν
Sambrook *et al.*, 2011 [16]	ν	ν	x	ν	x	ν	x	ν	ν
Brozek *et al.*, 2016 [17]	x	ν	ν	ν	x	x	ν	ν	ν
Peng *et al.*, 2016 [18]	ν	x	ν	x	ν	ν	ν	ν	x
Cobden *et al.*, 2019 [19]	x	ν	x	ν	x	x	ν	ν	ν
van Geel *et al.*, 2018 [20]	ν	ν	ν	ν	x	ν	x	ν	ν
Wang *et al.*, 2019 [21]	x	x	x	ν	x	ν	x	x	ν

Abtani *et al.*, 2020 [22]	x	v	v	x	x	x	v	v	v
Boonen *et al.*, 2011 [23]	v	v	v	v	v	v	v	x	v
Prieto-Alhambra *et al.*, 2014 [24]	v	x	v	v	v	v	v	x	x
Sing *et al.*, 2018 [25]	x	v	v	v	x	x	x	v	v
Nordstrom *et al.*, 2017 [26]	x	x	v	v	x	v	x	v	v
Center *et al.*, 2011 [27]	v	x	v	v	v	v	x	v	v
Degli Esposti *et al.*, 2012 [28]	x	x	v	v	v	x	v	v	v
Han *et al.*, 2020 [29]	v	x	v	v	v	x	v	x	x
Hsu *et al.*, 2019 [30]	x	v	x	v	v	v	x	v	v
Lebanon *et al.*, 2019 [31]	v	x	x	v	v	x	x	x	v
Chen *et al.*, 2020 [39]	x	v	v	v	v	x	v	v	v
Gonzales-Quevedo *et*	v	x	v	v	x	v	x	x	v

al., 2019 [32]									
Rotman-Pikielny *et al.*, 2018 [33]	ν	x	x	ν	x	x	ν	x	ν
Shen *et al.*, 2014 [34]	x	ν	ν	ν	ν	ν	ν	ν	ν
Palacios *et al.*, 2015 [35]	ν	x	ν	ν	ν	ν	ν	ν	ν
Osaki *et al.*, 2012 [36]	ν	x	ν	ν	ν	x	ν	ν	ν
Lee *et al.*, 2013 [38]	x	x	ν	ν	ν	ν	ν	ν	ν
Liu *et al.*, 2019 [37]	ν	ν	x	ν	ν	x	ν	ν	ν
Makras *et al.*, 2020 [40]	ν	ν	x	ν	ν	ν	ν	x	x

* arbitrary set as >1,000 for retrospective studies and >250 patients for randomized prospective studies;
x – not applicable; ν – characteristic found in the mentioned study;
